# *Pseudomonas aeruginosa* Polynucleotide Phosphorylase Contributes to Ciprofloxacin Resistance by Regulating PrtR

**DOI:** 10.3389/fmicb.2019.01762

**Published:** 2019-07-30

**Authors:** Zheng Fan, Hao Chen, Mei Li, Xiaolei Pan, Weixin Fu, Huan Ren, Ronghao Chen, Fang Bai, Yongxin Jin, Zhihui Cheng, Shouguang Jin, Weihui Wu

**Affiliations:** ^1^State Key Laboratory of Medicinal Chemical Biology, Key Laboratory of Molecular Microbiology and Technology of the Ministry of Education, Department of Microbiology, College of Life Sciences, Nankai University, Tianjin, China; ^2^Meishan Product Quality Supervision and Inspection Institute and National Pickle Quality Inspection Center, Meishan, China; ^3^Department of Molecular Genetics and Microbiology, College of Medicine, University of Florida, Gainesville, FL, United States

**Keywords:** *Pseudomonas aeruginosa*, polynucleotide phosphorylase, ciprofloxacin resistance, PrtR, pyocins

## Abstract

*Pseudomonas aeruginosa* is an opportunistic bacterial pathogen that causes various acute and chronic infections. It is intrinsically resistant to a variety of antibiotics. However, production of pyocins during SOS response sensitizes *P. aeruginosa* to quinolone antibiotics by inducing cell lysis. The polynucleotide phosphorylase (PNPase) is a conserved phosphate-dependent 3′–5′ exonuclease that plays an important role in bacterial response to environmental stresses and pathogenesis by influencing mRNA and small RNA stabilities. Previously, we demonstrated that PNPase controls the type III and type VI secretion systems in *P. aeruginosa*. In this study, we found that mutation of the PNPase coding gene (*pnp*) increases the bacterial resistance to ciprofloxacin. Gene expression analyses revealed that the expression of pyocin biosynthesis genes is decreased in the *pnp* mutant. PrtR, a negative regulator of pyocin biosynthesis genes, is upregulated in the *pnp* mutant. We further demonstrated that PNPase represses the expression of PrtR on the post-transcriptional level. A fragment containing 43 nucleotides of the 5′ untranslated region was found to be involved in the PNPase mediated regulation of PrtR. Overall, our results reveled a novel layer of regulation on the pyocin biosynthesis by the PNPase in *P. aeruginosa*.

## Introduction

*Pseudomonas aeruginosa* causes acute and chronic infections in immunocompromised patients (Balasubramanian et al., 2013). Emergence of drug-resistant *P. aeruginosa* strains greatly increases the difficulty of clinical treatment. Fluoroquinolone antibiotics have been used to treat *P. aeruginosa* infections (Andriole, 2005; Klodzinska et al., 2016). *P. aeruginosa* encodes multiple resistant determinants against fluoroquinolone antibiotics, such as multidrug efflux systems and pyocyanin (Subedi et al., 2018; Fan et al., 2019). However, chromosomally encoded pyocin biosynthesis genes increase the bacterial susceptibility to fluoroquinolone antibiotics (Brazas and Hancock, 2005; Sun et al., 2014; Chen et al., 2017). Ninety percent of *P. aeruginosa* strains produce pyocins, and each *P. aeruginosa* strain usually produces multiple types of the pyocins (Michel-Briand and Baysse, 2002; Ghequire and De Mot, 2014). Expression of the pyocin biosynthesis genes is activated by PrtN, while a λ CI homologous protein PrtR directly represses the transcription of *prtN* (Matsui et al., 1993). Genotoxic agents, including fluoroquinolone antibiotics and mitomycin-C, cause DNA damages, leading to the activation of RecA and subsequent SOS response. The activated RecA induces cleavage of PrtR, resulting in derepression of PrtN and production and release of pyocins, which are accompanied by lysis of the producer cells (Penterman et al., 2014).

Polynucleotide phosphorylase (PNPase) is a highly conserved exonuclease that degrades both RNA and ssDNA. In the presence of Mg^2+^ and inorganic phosphate (Pi), PNPase displays a 3′–5′ exoribonuclease activity. Meanwhile, PNPase can polymerize rNDP into RNA independent of a template. Thus, PNPase plays an important role in RNA metabolism in both prokaryotic and eukaryotic organisms (Cardenas et al., 2009, 2011; Cameron et al., 2018). In addition, in the presence of either Fe^3+^ or Mn^2+^ PNPase can polymerize dNDPs into ssDNA without a template. It also possesses a 3′–5′ exodeoxyribonuclease activity (Chou and Singer, 1971; Gillam and Smith, 1974; Beljanski, 1996). PNPase contains two PH domains at the N-terminus, forming a catalytic core and C-terminal RNA binding KH and S1 domains (Bermudez-Cruz et al., 2005; Briani et al., 2007; Fernandez-Ramirez et al., 2010). In addition, PNPase interacts with ribonuclease E, RNA helicase RhlB and enolase in certain species of Gram-negative bacteria, forming a RNA degradosome that plays an important role in mRNA decay (Carpousis, 2007; Nurmohamed et al., 2009). PNPase has been shown to be involved in bacterial responses to environmental stresses (Leszczyniecka et al., 2004; Cameron et al., 2018). In *Yersinia* and *Campylobacter jejuni*, PNPase is crucial for the growth at low temperatures (Haddad et al., 2009; Henry et al., 2012). In *Escherichia coli* and *Bacillus subtilis*, PNPase protects the bacterium against oxidative stresses mainly by promoting repair of oxidatively damaged DNA (Hayakawa et al., 2001; Cardenas et al., 2009, 2011; Wu et al., 2009) and contributes to bacterial survival upon UV radiation (Cardenas et al., 2009, 2011; Rath et al., 2012). PNPase has also been shown to be involved in the virulence of bacterial pathogens, including *Yersinia*, *Salmonellae*, and *Helicobacter pylori* (Rosenzweig et al., 2007; Hu and Zhu, 2015; Chen et al., 2016; Engman et al., 2016).

Previously, we demonstrated that PNPase is an essential gene in *P. aeruginosa*. Deletion of the KH and S1 domains results in downregulation of the type III secretion system and upregulation of the type VI secretion system (Chen et al., 2016). However, the role of PNPase in *P. aeruginosa* response to environmental stresses, such as antibiotics remains unknown. Here in this study, we found that mutation of the *pnp* increases the bacterial tolerance to fluoroquinolone antibiotics due to downregulation of the pyocin biosynthesis genes. We further demonstrated that the 5′-untranslated region (5′-UTR) of the *prtR* mRNA is involved in the PNPase mediated translational repression. Therefore, our results revealed a novel regulatory mechanism of pyocin production and the related bacterial resistance against ciprofloxacin.

## Materials and Methods

### Bacterial Strains, Growth Conditions, Plasmids and Primers

The bacterial strains, plasmids and primers used in this study were listed in Table 1 (Furste et al., 1986; Hoang et al., 1998; Choi and Schweizer, 2006; Sun et al., 2014; Chen et al., 2016, 2017). All bacterial strains were cultured in Luria–Bertani (LB) broth (5 g/L Nacl, 5 g/L yeast extract and 10 g/L tryptone, pH 7.4) at 37^∘^C with agitation at 200 rpm. All chromosomal gene mutations were generated as described previously (Hoang et al., 1998).

**TABLE 1:** Bacterial strains, plasmids and primers used in this study.

**Strain/Plasmid/Primer**	**Description**	**Source/Purpose**
***P. aeruginosa***		
PAK	Wild type strain of *Pseudomonas aeruginosa*	David Bradley
Δ*KH-S1*	PAK with *pnp* (KH and S1) deletion	Chen et al., 2016
Δ*KH-S1* /Tn7T-*pnp*	PAKΔ*KH-S1* with *pnp* inserted on chromosome with mini-Tn7T insertion;	Chen et al., 2016
PAKΔPA0614	PAK deleted of PA0614	This study
PAKΔPA0629	PAK deleted of PA0629	This study
PAKΔ*prtN*	PAK deleted of *prtN*	This study
Δ*KH-S1*ΔPA0614	PAKΔ*KH-S1* deleted of PA0614	This study
Δ*KH-S1*ΔPA0629	PAKΔ*KH-S1* deleted of PA0629	This study
Δ*KH-S1*Δ*prtN*	PAKΔ*KH-S1* deleted of *prtN*	This study
PAK/pMMB67EH	PAK containing plasmid pMMB67EH	This study
Δ*KH-S1*/pMMB67EH	PAKΔ*KH-S1* containing plasmid pMMB67EH	This study
PAK/pMMB67EH-*prtR*	PAK containing plasmid pMMB67EH-*prtR*	Chen et al., 2017
**Plasmid**		
pEX18Tc	Gene replacement vector; Tc^r^, *oriT*^+^, *sacB*^+^	Hoang et al., 1998
pUC18T-mini-Tn7T-Tc	mini-Tn7 base vector from insertion into chromosome attTn7 site; Tc^r^	Choi and Schweizer, 2006
pUC18T-mini-Tn7T-P*prtR*-lacZ	*prtR* promoter of PAK fused to promoterless *lacZ* on pUC18T-mini-Tn7T	Sun et al., 2014
pMMB67EH	Expression vector with tac promoter;Ap^r^	Furste et al., 1986
pUCP20 (no promoter)	*Escherichia-Pseudomonas* shuttle vector; no promoter; Amp^r^	This study
pUCP20(no promoter) -pRkaraRed(43)-PrtR-His	6 × His-tagged PrtR driven by the P_BAD_ promoter with 43 bp of the 5′-UTR sequence on pUCP20(no promoter)	This study
pUCP20(no promoter) -pRkaraRed(15)-PrtR-His	6 × His-tagged PrtR driven by the P_BAD_ promoter with 15 bp of the 5′-UTR sequence on pUCP20(no promoter)	This study
pUCP20(no promoter) -pRkaraRed(43)-GFP	GFP driven by the P_BAD_ promoter with 43 bp of the 5′-UTR sequence on pUCP20(no promoter)	This study
pUCP20(no promoter) -pRkaraRed(15)-GFP	GFP driven by the P_BAD_ promoter with 15 bp of the 5′-UTR sequence on pUCP20(no promoter)	This study
**Primer**	**Sequence (5′→3′)**	**Function**
PA0636-RT-S	TGGAAGACCCGGCAGAAG	RT-PCR
PA0636-RT-AS	CGTTGAGCTTGGACAGATCCT	RT-PCR
PA0614-RT-S	CGCTGCCTGCCAAGGA	RT-PCR
PA0614-RT-AS	ATCAGTACCCAGAGCGGCATT	RT-PCR
PA0629-RT-S	GTGGAGAACCTCAATTACAG	RT-PCR
PA0629-RT-AS	TAGGTGTTGTCGGCAATC	RT-PCR
*prtR*-RT-S	GATGCGCAACCTGAAGCA	RT-PCR
*prtR*-RT-AS	TGAATGGTGTTCTGCGAAACC	RT-PCR
*prtN*-RT-S	CGACGATAGCCACAAG	RT-PCR
*prtN*-RT-AS	GGATGCGATGCTGTC	RT-PCR
*lexA*-RT-S	AATCCCGCCTTCTTCAAT	RT-PCR
*lexA*-RT-AS	AATGCCGATGTCCTTCAT	RT-PCR
*recA*-RT-S	ATATCAAGAACGCCAACT	RT-PCR
*recA*-RT-AS	TAGAACTTCAGTGCGTTA	RT-PCR
BamHI-P*prtR*-*lacZ*-S^#^	CGCGGATCC GAGCCAGGACCAGTTCGTTGGC	Transcriptional fusion
HindIII-*lacZ*-AS	ATTATAAAGCTT TTATTTTTGACACCAGACCAACTGG	Transcriptional fusion
SacI-P_BAD_-S	CCAAGAGCTC TTATGACAACTTGACGGC	Translational fusion
HindIII-*prtR*-AS	ATTATAAAGCTT TCAGTGGTGGTGGTGGTGGTGACCTCCCC GCACCAGGGACGGGCCGC	Translational fusion
XhoI- *prtR*(43)-GFP S	CCGCTCGAG TAGGCTCTTTACAGAAAATCCATCGGTCTGTAGA TTGCCGAGCATGAGTAAAGGAGAAGAACTTTTCACTG	Translational fusion
XhoI- *prtR*(15)-GFP S	CCGCTCGAG TGTAGATTGCCGAGCATGAGTAAAGGAGAAGAA CTTTTCACTG	Translational fusion
HindIII –GFP-AS	CCCAAGCTT TTATTTGTATAGTTCATCCATGCCATG	Translational fusion

*^#^The endonuclease cutting sites are underlined.*

### Minimum Inhibitory Concentration and Survival Assay

Minimum inhibitory concentrations were determined by the twofold serial dilution method as described previously (Fan et al., 2019). Overnight bacterial cultures were diluted 1:50–1:100 in LB and cultured at 37^∘^C until the OD_600_ reached 0.8–1.0. The bacterial concentration was adjusted to 1 × 10^5^ CFU/ml and 200 μl of the bacteria was added to each well of a 96-well plate (Corning). The plate was incubated for 24 h at 37^∘^C without agitation. The Minimum inhibitory concentration was recorded as the lowest concentration of antibiotic that inhibited visible growth. For the survival assay, bacteria were grown to an OD_600_ of 1.0 at 37^∘^C. Then the bacteria were treated with ciprofloxacin at indicated concentrations at 37^∘^C with agitation at 200 rpm. The numbers of live cells before and after antibiotic treatment were determined by serial dilution and plating assay.

### RNA Extraction, Reverse Transcription, and Quantitative RT-PCR

Overnight bacterial cultures were diluted 1:50–1:100 into fresh LB with and without 0.016 μg/ml ciprofloxacin and grown to an OD_600_ of 0.8–1.0. Total RNA was isolated with an RNeasy Mini kit (Tiangen Biotech, Beijing, China) and cDNA was synthesized with a PrimeScript Reverse Transcriptase (TaKaRa, Dalian, China). 1 μg RNA was used for reverse transcription. In the quantitative RT-PCR experiment, the cDNA was mixed with specific forward and reverse primers and the SYBR Premix Ex Taq^TM^ II (TaKaRa). The CFX Connect Real-Time system (Bio-Rad, United States) was used to perform the quantitative RT-PCR. *rpsL*, which encodes the 30S ribosomal protein S12 was used as an internal control.

### Western Blotting

Samples from the same number of bacterial cells were loaded onto 10 or 12% sodium dodecyl sulfate-polyacrylamide (SDS-PAGE) gel. Then the proteins were transferred onto a polyvinylidene difluoride (PVDF) membrane and probed with a GFP antibody or a mouse monoclonal antibody against the 6 × His tag (1:2000; Cell Signaling Technology, United States) at room temperature for 1–2 h or overnight at 4^∘^C. Then the PVDF membrane was washed with 1 × phosphate-buffered saline (1 × PBS, 5.4 mM KCl, 20 mM Na_2_HPO_4_, 274 mM NaCl, 4 mM KH_2_PO_4_, pH 7.4) containing 2% 24 times. Next, the PVDF membrane was incubated with an anti-rabbit IgG (1: 2,000; Promega, United States) at room temperature for 1.5 h. Signals were detected by an ECL Plus kit (Millipore). The signals were visualized by a Bio-Rad molecular imager (ChemiDocXRS). The RNA polymerase α subunit RpoA was used as a loading control (with an antibody from Biolegend).

### Promoter Activity Assay

The promoter region of the *prtR* gene was amplified by PCR with the primers shown in Table 1. The PCR product was fused with the coding sequence of *lacZ*. The P*_prtR_*-*lacZ* fusion was inserted into the chromosome of *P. aeruginosa* strains via a miniTn7 vector (Choi and Schweizer, 2006). To measure the expression level of LacZ, the bacteria were grown to an OD_600_ of 0.5, and then treated with ciprofloxacin at indicated concentrations for 3 h. The β-galactosidase activities were measured as described previously (Weng et al., 2016). Briefly, each sample (0.5 ml bacteria) was collected by centrifugation and resuspended in 1.5 ml Z buffer (60 mM NaH_2_PO_4_, 60 mM Na_2_HPO_4_, 1 mM MgSO_4_, 10 mM KCl and 50 mM β-mercaptoethanol, pH 7.0). 0.5 ml of the suspension was mixed with 10 μl 0.1% SDS (BBI Life Sciences, Shanghai, China) and 10 μl chloroform (BBI Life Sciences, Shanghai, China), and then vortexed for 10–15 s. The remaining 1 ml was used for OD_600_ measurement. 100 μl ONPG (40 mg/ml; Sigma, United States) was added to each sample, followed by incubation at 37^∘^C. When the color turned into light yellow, 0.5 ml 1 M Na_2_CO_3_ was added to the mixture to stop the reaction. OD_420_ was measured, and the time was recorded. The β-galactosidase activity (Miller units) was calculated as (1000 × OD_420_)/(T × V × OD_600_). T, reaction time (minute); V, bacteria volume (ml).

## Results

### PNPase Influences the Bacterial Resistance to Ciprofloxacin

**FIGURE 1:**
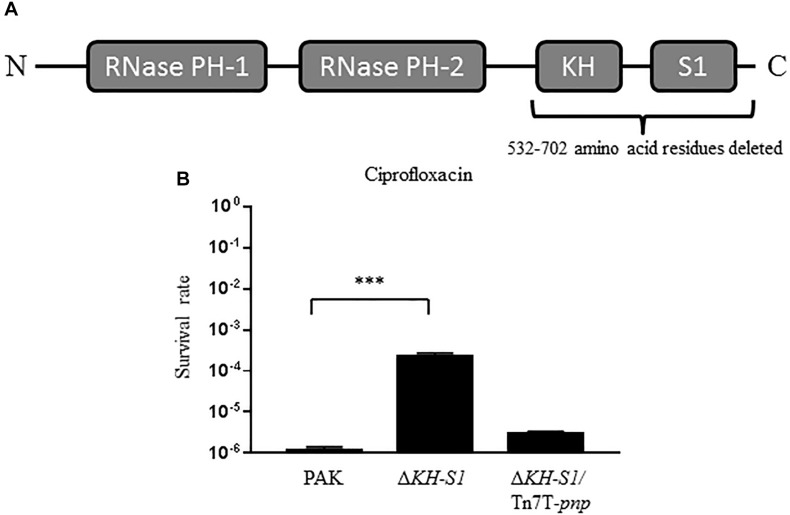
Bacterial survival rates under the treatment of ciprofloxacin. **(A)** Domains of the PNPase of *Pseudomonas aeruginosa*. **(B)** PAK, Δ*KH-S1* mutant and the complemented strain (Δ*KH-S1*/Tn7T-*pnp*) were grown to an OD_600_ of 1.0 at 37^∘^C and treated with 0.16 μg/ml ciprofloxacin for 6 h. At indicated time points, the bacterial survival rates were determined by serial dilution and plating assay. ^∗∗∗^*p* < 0.001 by Student’s *t*-test.

To test the role of PNPase in antibiotic resistance of *P. aeruginosa*, we determined the MICs of various antibiotics against wild type PAK and an isogenic mutant with the deletion of the KH-S1 domains of PNPase (Δ*KH-S1*) (Figure 1A; Chen et al., 2016). The two strains displayed similar levels of resistance (MICs) to most of the tested antibiotics, including erythromycin, carbenicillin and gentamicin. However, the MICs of ciprofloxacin and ofloxacin were increased four and two fold in the Δ*KH-S1* mutant, respectively (Table 2). Complementation with a *pnp* gene restored the bacterial susceptibility (Table 2). Consistent with the MIC test results, in the presence of 0.16 μg/ml (1 × MIC) ciprofloxacin, deletion of the KH-S1 domains increased the bacterial survival rate by approximately 100-fold, which was restored by complementation with a *pnp* gene (Figure 1B).

**TABLE 2:** Bacterial susceptibilities to antibiotics.

	**MIC (μg/ml)**				
**Strain**	**Ciprofloxacin**	**Ofloxacin**	**Carbenicillin**	**Erythromycin**	**Gentamicin**
PAK	0.16	1.5	150	125	0.625
Δ*KH-S1*	0.64	3	150	125	0.625
Δ*KH-S1*/Tn7T-*pnp*	0.16	1.5	150	125	–

*“–” Indicates that the complemented strain is resistant to gentamicin due to the miniTn7T insertion.*

### Downregulation of Pyocin Biosynthesis Genes Contributes to the Increased Resistance to Ciprofloxacin in the ΔKH-S1 Mutant

**FIGURE 2:**
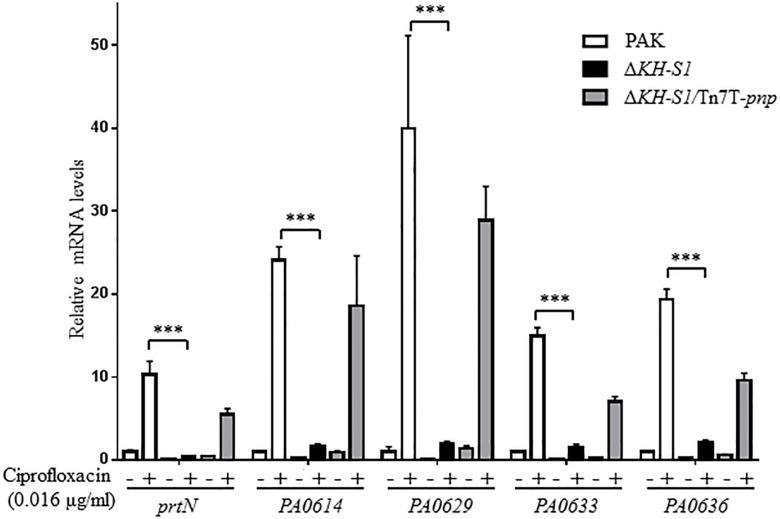
Expression levels of pyocin biosynthesis genes in the ΔKH-S1 mutant. PAK, Δ*KH-S1* and the complemented strain were grown to an OD_600_ of 0.8–1.0 at 37^∘^C with or without 0.016 μg/ml ciprofloxacin, followed by RNA extraction. The mRNA levels of *prtN*, *PA0614*, *PA0629*, *PA0633*, and *PA0636* were determined by real-time PCR with *rpsL* as the internal control. ^∗∗∗^*p* < 0.001 by Student’s *t*-test.

In our previous transcriptome analysis of the Δ*KH-S1* mutant, no alternation was observed on the expression of the multidrug efflux system genes, whereas the pyocin biosynthesis genes were downregulated (Chen et al., 2016). Due to the role of pyocins in the bacterial susceptibility to ciprofloxacin (Brazas and Hancock, 2005; Sun et al., 2014; Chen et al., 2017), we verified the expression levels of the R-type (*PA0614*) and F-type pyocins (*PA0629*, *PA0633*, and *PA0636*) genes by real time PCR (Nakayama et al., 2000; Michel-Briand and Baysse, 2002). Due to the difference in the MICs of ciprofloxacin to wild type PAK and the Δ*KH-S1* mutant, we treated both strains with 0.016 μg/ml ciprofloxacin (1/10 MIC to PAK), which did not affect the growth of both strains. In the presence or absence of ciprofloxacin, the mRNA levels of the pyocin biosynthesis genes were lower in the Δ*KH-S1* mutant than those in wild type PAK. Complementation with a *pnp* gene restored the mRNA levels in the Δ*KH-S1* mutant (Figure 2). In PAK, the resistance to ciprofloxacin was increased upon deletion of *prtN*, *PA0614*, and *PA0629*, which encode the transcriptional activator for the pyocin biosynthesis genes, a holin- and a lysozyme-like protein, respectively (Table 3). However, deletion of those genes in the Δ*KH-S1* mutant did not further increase the resistant level (Table 3), indicating that the repression of pyocin biosynthesis genes might result in the increased resistance to ciprofloxacin.

**TABLE 3:** Bacterial susceptibilities to ciprofloxacin.

**Strain**	**MIC (μg/ml)**
PAK	0.16
ΔPA0614	0.32
ΔPA0629	0.32
Δ*prtN*	0.32
Δ*KH-S1*	0.64
Δ*KH-S1*ΔPA0614	0.64
Δ*KH-S1*ΔPA0629	0.64
Δ*KH-S1*Δ*prtN*	0.64
PAK/pMMB67EH	0.16
PAK/pMMB67EH-*prtR*-His	0.64
Δ*KH-S1*/pMMB67EH	0.64

### The PrtR Protein Level Is Increased in the ΔKH-S1 Mutant

**FIGURE 3:**
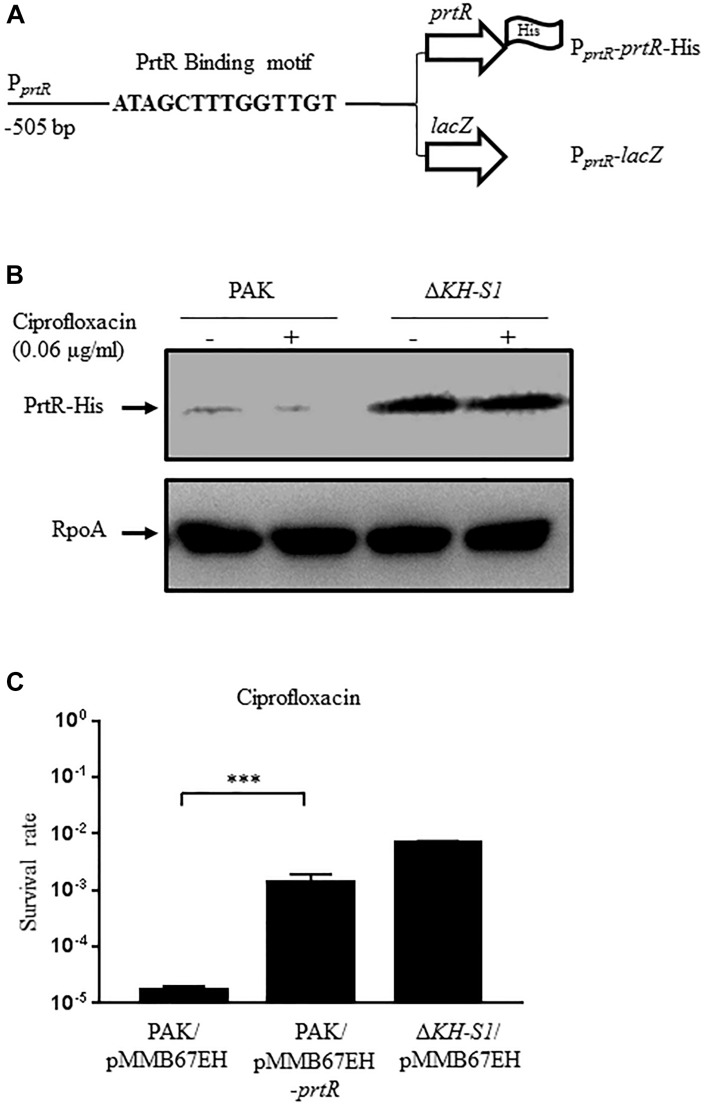
Expression of PrtR in the ΔKH-S1 mutant. **(A)** Fragments of the prtR promoter region fused with the prtR-His or a promoterless lacZ gene. **(B)** Protein levels of PrtR-His in PAK and the Δ*KH-S1* mutant carrying the P*_prtR_*-*prtR*-His on the bacterial chromosome. The bacterial cells were grown to an OD_600_ of 1.0, and then incubated with or without 0.06 μg/ml ciprofloxacin for 1 h. The PrtR-His levels were determined by Western blotting. RpoA was used as the loading control. **(C)** PAK containing an empty vector or the *prtR* overexpression plasmid was grown to an OD_600_ of 1.0 and treated with 0.16 μg/ml ciprofloxacin for 6 h. At indicated time points, the bacterial survival rate was determined by serial dilution and plating. ^∗∗∗^*p* < 0.001 by Student’s *t*-test.

PrtR directly represses the transcription of *prtN* that encodes the transcriptional activator of the pyocin biosynthesis genes (Matsui et al., 1993). Since the mRNA level of *prtN* was lower in the Δ*KH-S1* mutant (Figure 2), we suspected that the PrtR protein level might be higher in the Δ*KH-S1* mutant. To test the protein level of PrtR, we utilized a C-terminal 6 × His-tagged *prtR* driven by its native promoter (designated as P*_prtR_*-*prtR*-His) (Figure 3A; Sun et al., 2014). Indeed, the PrtR-His level was higher in the Δ*KH-S1* mutant than that in PAK in the presence or absence of ciprofloxacin (Figure 3B). In addition, overexpression of *prtR* in PAK increased the MIC of ciprofloxacin by fourfold and enhanced the survival rate in the presence of ciprofloxacin to the similar level as that of the Δ*KH-S1* mutant (Figure 3C and Table 3). These results suggest that the increased resistance to ciprofloxacin is likely due to the higher protein level of PrtR in the Δ*KH-S1* mutant.

### PNPase Affects the Expression of PrtR at the Post-transcription Level Through Its 5*′*-UTR

**FIGURE 4:**
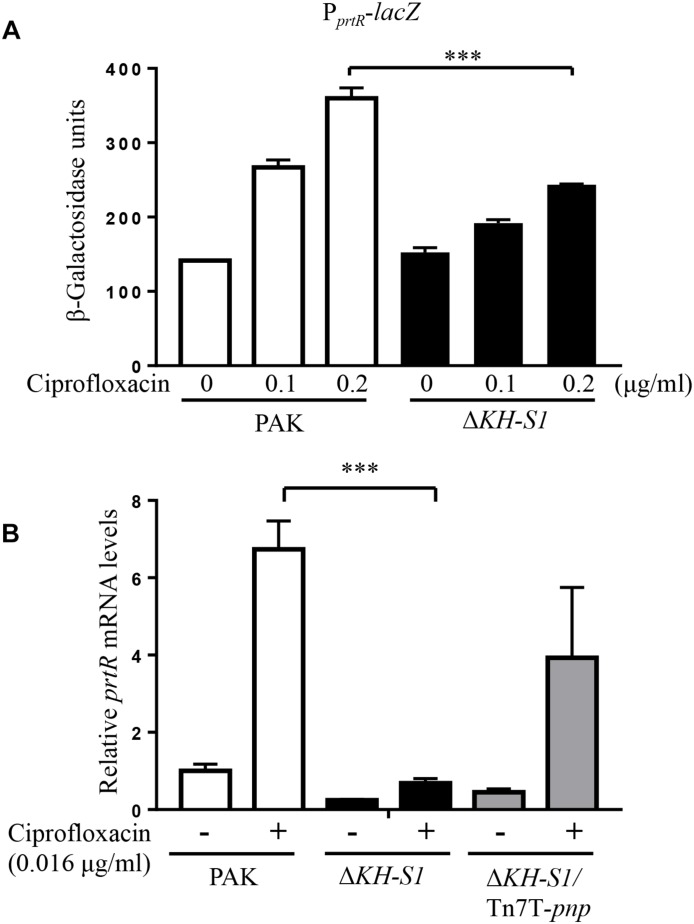
The promoter activity and mRNA level of prtR in the ΔKH-S1 mutant. **(A)** Expression of P*_prtR_*-*lacZ* in PAK and the Δ*KH-S1* mutant. The bacteria were grown to an OD_600_ of 0.5, and then treated with ciprofloxacin at indicated concentrations for 3 h, followed by the β–galactosidase assay. ^∗∗∗^*p* < 0.001 by Student’s *t*-test. **(B)** PAK, Δ*KH-S1* and the complemented strain were grown to an OD_600_ of 0.8–1.0 at 37^∘^C with or without 0.016 μg/ml ciprofloxacin. The mRNA levels of *prtR* were determined by real-time PCR with *rpsL* as the internal control. ^∗∗∗^*p* < 0.001 by Student’s *t*-test.

To understand the mechanism of the increased PrtR level, we examined the promoter activity by utilizing a transcriptional fusion of *lacZ* reporter gene with the promoter of *prtR* (P*_prtR_*-*lacZ*). The presence of ciprofloxacin induced the *lacZ* expression in wild type PAK, however, the *lacZ* expression levels in the Δ*KH-S1* mutant were lower than those in PAK in the presence of the same concentrations of ciprofloxacin (Figure 4A). Consistent with the above results, the mRNA level of *prtR* was lower in the Δ*KH-S1* mutant (Figure 4B), which might be due to an auto-repression of PrtR (Sun et al., 2014). Nevertheless, this result indicates that the upregulation of PrtR in the Δ*KH-S1* mutant might be mediated through a post-transcriptional mechanism.

**FIGURE 5:**
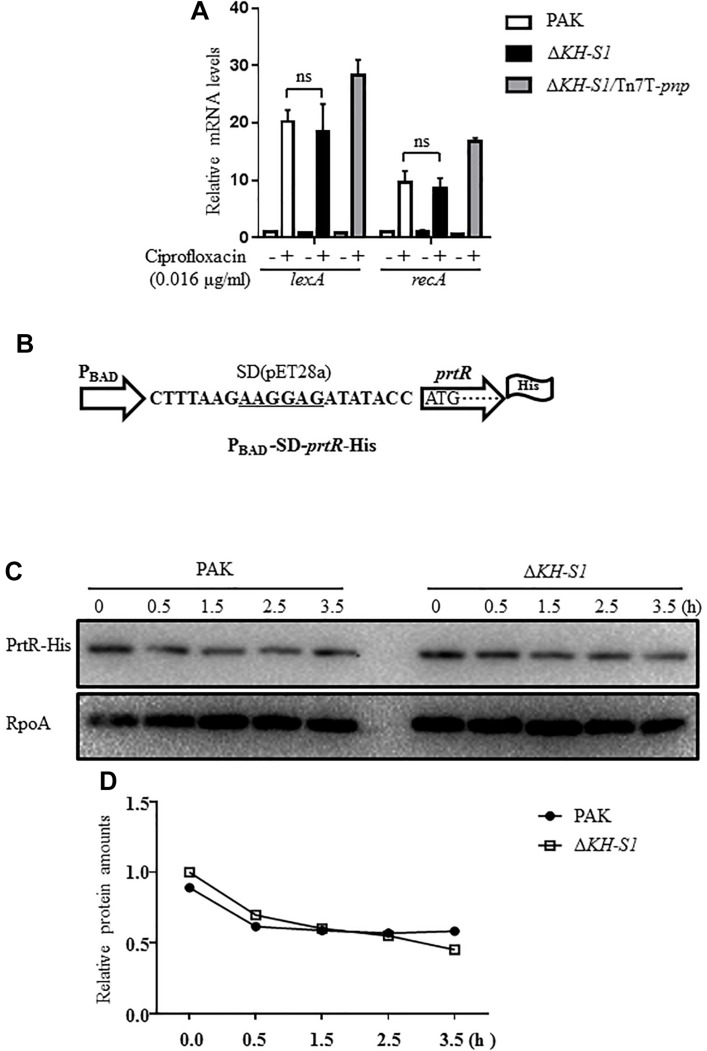
PrtR protein stabilities in PAK and the ΔKH-S1 mutant. **(A)** PAK, ΔKH-S1 and the complemented strain were grown to an OD600 of 0.8–1.0 at 37^∘^C with or without 0.016 μg/ml ciprofloxacin. The mRNA levels of *lexA* and *recA* were determined by real-time PCR with *rpsL* as the internal control. ns, not significant by Student’s *t*-test. **(B)** The C-terminal 6 × His-tagged *prtR* is driven by an inducible P_BAD_ promoter with an exogenous ribosome binding site (designated as P_BAD_-SD-*prtR*-His). The ribosome binding sequence was underlined. **(C,D)** Strains carrying the P_BAD_-SD-*prtR*-His were grown to an OD_600_ of 0.6–0.8 at 37^∘^C, followed by induction with 0.2% arabinose for 1.5 h. Then, 500 μg/ml chloramphenicol and 0.016 μg/ml ciprofloxacin were added to the medium. At the indicated time points, bacterial cells of each strain were collected and the levels of PrtR-His were determined by Western blotting. RpoA was used as the loading control. The relative intensity of each band was quantified by ImageJ.

Previous studies demonstrated that the stability of PrtR is regulated by RecA in response to genotoxic agents (Sun et al., 2014). Treatment with ciprofloxacin induced similar expression levels of *recA* and *lexA* in the Δ*KH-S1* mutant and PAK, indicating a similar level of SOS response (Figure 5A). To examine the PrtR protein stability, we constructed a C-terminal 6 × His-tagged PrtR driven by an inducible P_BAD_ promoter with an exogenous ribosome binding site from the vector pET28a, resulting in P_BAD_-SD-*prtR*-His (Figure 5B). In the absence of ciprofloxacin, the levels of the PrtR-His were similar in the Δ*KH-S1* mutant and PAK. Treatment with ciprofloxacin resulted in a similar degradation rate of the PrtR-His in both strains (Figures 5C,D).

**FIGURE 6:**
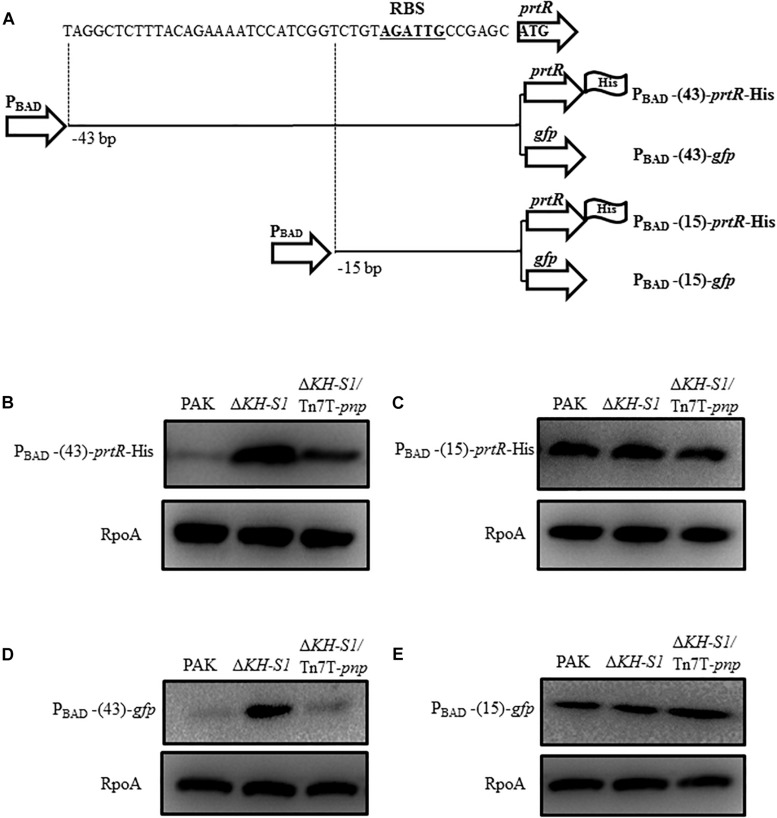
Translational regulation of prtR by the PNPase. **(A)** Structures of the 6 × His-tagged *prtR* fusions. P_BAD_-43-*prtR*-His and P_BAD_-15-*prtR*-His represent 6 × His-tagged *prtR* driven by the P_BAD_ promoter with 43 and 15 bp of the 5′-UTR sequence of the *prtR* gene, respectively. The *prtR* open reading frame was replaced by a *gfp* gene, resulting in P_BAD_-43-*gfp* and P_BAD_-15-*gfp*. The potential ribosome binding site (RBS) was shown in bold underlined letters. Strains containing the *prtR*-His or *gfp* expression plasmid were grown to an OD_600_ of 1.0 and then induced with 0.2% arabinose for 1.5 h. Protein levels of PrtR **(B,C)** and GFP **(D,E)** were determined by Western blotting. RpoA was used as the loading control.

We then examined whether the translation of the *prtR* mRNA is affected in the Δ*KH-S1* mutant. Since the 5′ untranslated region (5′-UTR) of a mRNA is usually involved in the translational regulation, we constructed a 6 × His-tagged *prtR* driven by an exogenous P_BAD_ promoter with 43 bp of the *prtR* 5′-UTR sequence (Figure 6A). The translation of the PrtR was higher in the Δ*KH-S1* mutant (Figure 6B). To identify the region involved in the post-transcriptional regulation, we reduced the 5′-UTR sequence to 15 bp, resulting in P_BAD_-15-*prtR*-His (Figure 6A). From this construct, similar levels of PrtR-His were observed in the Δ*KH-S1* mutant and wild type PAK (Figure 6C). As the coding region might be involved in the translational regulation, we replaced the *prtR* coding sequence with a *gfp* gene, resulted in P_BAD_-43-*gfp* and P_BAD_-15-*gfp*, respectively (Figure 6A). Fusion with the 43 bp 5′-UTR of *prtR* resulted in higher GFP level in the Δ*KH-S1* mutant, which was restored by complementation with a *pnp* gene (Figure 6D). However, reduction of the 5′-UTR to 15 bp resulted in similar levels of GFP (Figure 6E). These results suggest that the 5′-UTR of the *prtR* mRNA might be involved in the PNPase mediated post-transcriptional regulation of PrtR.

## Discussion

In this study, we found that deletion of the KH-S1 domains of the PNPase increased the bacterial resistance to fluoroquinolone antibiotics. We further demonstrated that the PrtR level is increased in the Δ*KH-S1* mutant, which reduces the PrtN expression, resulting in downregulation of the pyocin biosynthesis genes in the presence of ciprofloxacin.

The PNPase is a conserved exoribonuclease that degrades single stranded RNA. It contains two N-terminal PH domains that possess the ribonuclease activity, and C-terminal KH and S1 domains that are involved in the binding of RNAs. The PNPase plays an important role in the maturation of rRNAs and tRNAs. Besides, the PNPase has been shown to control gene expression through sRNAs. In *Salmonella typhimurium*, Hfq independent sRNAs CsrB, CsrC, and CopA are initially cleaved by RNase E, followed by degradation by PNPase (Viegas et al., 2007). In *E. coli*, PNPase degrades the sRNAs SgrS, GlmY, MicA, and RyhB when they are not bond to Hfq (Andrade et al., 2012). Meanwhile, PNPase also increases the stability of certain Hfq-bond sRNAs (Bandyra et al., 2016). For instance, deletion of *pnp* in *E. coli* resulted in reduced level of ArcZ, a negative regulator of *mutS*. Consequently, upregulation of *mutS* in the *pnp* mutant decreases bacterial spontaneous mutation rate (Chen and Gottesman, 2017).

Previously, we demonstrated that PNPase regulates type VI secretion system through degradation of the sRNAs RsmY and RsmZ (Chen et al., 2016). In this study, we found that a 43-nucleotide 5′-UTR of the *prtR* mRNA is required for the PNPase mediated translational repression. Reduction of the 5′-UTR to 15-nucleotide resulted in the similar levels of the PrtR protein in the Δ*KH-S1* mutant and wild type strain. The 5′-UTR might control gene expression through several mechanisms. For example, formation of a hairpin structure might block the ribosome binding site. PNPase might affect the secondary structure by recruiting an endonuclease. Another possibility is that a sRNA might anneal to the 5′-UTR, which alters the secondary structure or directly blocks the ribosome binding site. In addition, PNPase might directly bind to an mRNA though its KH-S1 domains, which affects the translation. To examine whether PNPase can directly bind to the 5′-UTR of the *prtR* mRNA, we performed an RNA electrophoretic mobility shift assay. However, no interaction was observed (data not show). It might be possible that another protein is required to facilitate the interaction. Further studies are needed to elucidate the regulatory mechanism.

Pyocins are chromosomally encoded bacteriocins produced by most of *P. aeruginosa* strains. Production and release of pyocins under environmental stresses such as the presence of genotoxic agents might provide an advantage in the competition against other bacteria (Michel-Briand and Baysse, 2002). A recent study revealed that R-type pyocins play an important role in the competition among various *P. aeruginosa* strains during the infection of cystic fibrosis patients (Oluyombo et al., 2019). In addition, when pyocins are released through cell lysis, the liberated chromosomal DNA and other components function as the matrix for biofilm formation (Turnbull et al., 2016). However, for the individual pyocins producer cells, the release of pyocins leads to cell death. Therefore, the production of pyocins should be under a tight control. Our study here revealed a novel post-transcriptional regulation on the key regulator PrtR. Further studies are needed to elucidate the molecular details of the regulatory mechanism and the signaling pathway.

## Data Availability

All datasets generated for this study are included in the manuscript and/or the supplementary files.

## Author Contributions

ZF, WW, and SJ conceived and designed the experiments. ZF, HC, ML, XP, WF, HR, and RC performed the experiments. YJ, WW, FB, ZC, and SJ analyzed the data. ZF, WW, and SJ wrote the manuscript.

## Conflict of Interest Statement

The authors declare that the research was conducted in the absence of any commercial or financial relationships that could be construed as a potential conflict of interest.
